# High Incidence of Group B Streptococcal Infection in Infants Born to HIV-Infected Mothers

**DOI:** 10.3201/eid1803.111630

**Published:** 2012-03

**Authors:** Tessa Goetghebuer, Catherine Adler, Cristina Epalza, Jack Levy

**Affiliations:** Saint-Pierre University Hospital, Brussels, Belgium

**Keywords:** Streptococcus agalactiae, bacteria, group B streptococcal infection, GBS, HIV, pregnant women, infants, HIV/AIDS and other retroviruses, streptococci

**To the Editor:** In their cross-sectional study comparing group B streptococcus (GBS) carriage among HIV-infected and HIV-uninfected women in Malawi, Gray et al. found no differences in GBS carriage between both groups but found a higher carriage rate for HIV-infected women with high CD4 cell counts (*1*). They proposed that a GBS-specific immune defect might exist in HIV-infected pregnant women and suggested that this defect could be blurred by competitive exclusion of GBS as a consequence of changes in microbial flora at lower CD4 counts.

We recently reported an increased incidence of neonatal GBS sepsis in HIV-exposed uninfected (HEU) infants born in Belgium, compared with the general population (*2*). In our cohort, the risk for GBS infection was 20× higher in HEU infants than in infants born to HIV-uninfected mothers. Moreover, the episodes of GBS sepsis in HEU infants, compared with the general population, were more severe and mostly of late onset. We are currently looking prospectively at GBS carriage in HIV-infected and control uninfected pregnant women to learn whether our observation can be explained by a higher carriage rate in HIV-infected women or by increased susceptibility of HEU infants to this capsulated bacteria. The latter hypothesis would be in line with the higher susceptibility of HEU children to other types of severe infections, as has been described in several studies from sub-Saharan Africa and Latin America (*3**–**5*).

The incidence of GBS sepsis in HIV-exposed infants born in Africa is unknown. In addition to the need for further investigation of anti-GBS immunity in HIV-infected pregnant women, we believe that studies comparing the incidence of neonatal GBS sepsis in HEU and HIV-unexposed infants are warranted. If the increased risk for GBS sepsis is confirmed, prophylaxis should be implemented in the population concerned.
